# Supplementary material for: Improving extractive document summarization with sentence centrality
Source: PLoS One. 2022 Jul 22;17(7):e0268278. doi: 10.1371/journal.pone.0268278 (PMC9307201; doi:10.1371/journal.pone.0268278)
Supplement: S1 Appendix — (PDF) [file pone.0268278.s001.pdf]

Supporting information

S1 Appendix

ROUGE stands for Recall-Oriented Understudy for Gisting Evaluation. The calculation methods of ROUGE-N and ROUGE-L are presented below.

ROUGE-N is an n-gram recall between a candidate summary and a set of reference summaries. ROUGE-N is computed as follows:

$$ROUGE\text{-}N = \frac{\sum_{S \in \text{ReferemceSummaries}} \sum_{gram_n \in S} Count_{match}(gram_n)}{\sum_{S \in \text{ReferemceSummaries}} \sum_{gram_n \in S} Count(gram_n)},$$

where n stands for the length of the n-gram, $gram_n$ and $Count_{match}(gram_n)$ is the maximum number of n-grams co-occurring in a candidate summary and a set of reference summaries.

Given a reference summary X of length m and a candidate summary of length n, ROUGE-L can be computed as follows:

$$R_{lcs} = \frac{LCS(X,Y)}{m},$$

$$P_{lcs} = \frac{LCS(X,Y)}{n},$$

$$F_{lcs} = \frac{(1+\beta^2)R_{lcs}P_{lcs}}{R_{lcs}+\beta^2 P_{lcs}}.$$

$LCS(X,Y)$ is the length of a longest common subsequence of X and Y, $\beta = P_{lcs}/R_{lcs}$, F_{lcs} is the score of ROUGE-L.
